# Exploiting Laser-Ablation ICP-MS for the Characterization of Salt-Derived Bismuth Films on Screen-Printed Electrodes: A Preliminary Investigation

**DOI:** 10.3390/bios10090119

**Published:** 2020-09-09

**Authors:** Carlo Dossi, Gilberto Binda, Damiano Monticelli, Andrea Pozzi, Sandro Recchia, Davide Spanu

**Affiliations:** 1 Dipartimento di Scienze Teoriche ed Applicate, Università degli Studi dell’Insubria, Via Dunant, 3, 22100 Varese, Italy; g.binda2@uninsubria.it; 2 Dipartimento di Scienza ed Alta Tecnologia, Università degli Studi dell’Insubria, Via Valleggio, 11, 21100 Como, Italy; Damiano.Monticelli@uninsubria.it (D.M.); andrea.pozzi@uninsubria.it (A.P.); sandro.recchia@uninsubria.it (S.R.); d.spanu1@uninsubria.it (D.S.)

**Keywords:** laser ablation ICP-MS, surface characterization, bismuth film electrodes, screen-printed electrodes, trace electroanalysis

## Abstract

The use of insoluble bismuth salts, typically BiPO_4_, is known to be a viable alternative to classical Bi^3+^ ion electrochemical reduction for the preparation of bismuth film electrodes (BiFE) on screen-printed electrodes. The freshly prepared electrodes are indefinitely stable, and the active bismuth film is simply formed by in situ reduction. Two aspects are still to be investigated, namely the bismuth distribution on the working electrode and the possible residual presence of the counteranion, namely phosphate. High-vacuum techniques such as electron microscopy or spectroscopy, which are commonly employed for this purpose, cannot be safely used: the bismuth surface is well-known to reconstruct and recrystallize under the electron beam in vacuum. Here, we demonstrate the suitability and the effectiveness of laser ablation ICP-MS (LA-ICP-MS, a technique that vaporizes and analyzes the surface material under flowing helium at atmospheric pressure) for the characterization of BiFE. Fast and stable measurements of bismuth and phosphorous distribution are achieved with the advantage of a minimum alteration of the sample surface, avoiding possible interferences. This investigation evidenced how, upon reductive activation, the bismuth film is distributed with a radial symmetry and the phosphate counteranion is completely absent on the working electrode surface.

## 1. Introduction

The pioneering works by Wang and Hocevar on bismuth [1,2] had the merit to draw considerable interest in this “green” element to prepare electroactive surface films as a low-toxicity alternative to conventional mercury films. In fact, bismuth shows evident analogies with mercury in forming alloys and adsorptive complexes with many metals [3]. Although many years have passed since the introduction of voltammetry and stripping for the detection of trace heavy metals, research on these electroanalytical techniques is still a hot topic, since many advantages may be seen compared to classical optical techniques, not simply their low cost. Interestingly, increased interest to “green” metal modifiers other than bismuth is also observed [4,5,6,7]. It is finally noteworthy that bismuth, thanks to its very low toxicity, will play a definite role for the electroanalytical studies of organic as well as biological molecules [8,9,10].

The preparation of an active bismuth film on a screen-printed electrode (BiFE-SPE) is generally carried out under the conventional electrochemical reduction of Bi^3+^ ion in aqueous electrolytes. This process is not a facile one, since it is reported to be sensitive to the nature of the bismuth precursor, the possible occurrence of kinetics limitations, and the sensitivity to the cleanliness of the carbon surface of the working electrode [11,12]. This wide range of experimental conditions leading to the optimal and active bismuth film may be rationalized on the basis of the rich chemistry of bismuth in aqueous solutions [13]. In this context, a decisive advantage was gained applying surface modification with polymers: this treatment permits to mitigate interferences and to improve the mechanical stability of the bismuth film [14,15]. As an additional benefit, some polymeric layers are reported to have a synergistic sensing effect (e.g., using carboxymethyl cellulose which is capable of chelating cadmium ions) [16].

An alternative and innovative approach was proposed by Brainina et al. [17]. This strategy involves the in situ formation of an insoluble bismuth salt (e.g., BiPO_4_) during the electrode preparation with the polymeric film. The bismuth film is then formed by reductive activation. However, the deposition of the insoluble salt precursor may be critical, and the following activation to the active bismuth surface may be complicated by liquid/solid phase kinetics. These conditions require a precise microscopical characterization of the bismuth film and the electrode surface, both before and after the electrochemical activation to ensure electrode performances. Two aspects are of main interest for their characterization: the bismuth distribution and the possible residual presence of the counteranion of the insoluble salt, namely phosphate.

A number of groups have used scanning and transmission electron microscopy [4,5,6,10,17,18,19] for the investigation of these features on bare and modified metal films on SPE, showing very different shapes and morphology. However, bismuth (or mercury) is a soft metal, and possible changes of the phase state of the thin film have been evidenced, due to the combined effect of high-vacuum and high-energy beam conditions [20]. Therefore, it should be taken into consideration the possibility that the observed shape and morphology of the metal surface may be profoundly dependent upon the experimental conditions of the high-vacuum experiment. Surface spectroscopies, primarily X-ray photoelectron spectroscopy (XPS), are also reported to be used to characterize metal films, as well as nanomodifiers, on screen-printed electrodes [21,22,23]. XPS is, in fact, very effective to probe the chemical composition of the outermost few nanometers of materials and the electrochemical activity strictly depends on the first atomic layers in contact with the electrolytic solution. However, no imaging information is likely to be obtained.

In this paper, the laser ablation ICP-MS technique has been proposed as a potential substitute (or complementary) technique of electron microscopy or surface spectroscopy for the investigation of the bismuth surface. Lasers have been used for the modification and functionalization of electrodes, and laser-induced forward transfer (LIFT) has emerged as a powerful physisorption method for the deposition of various materials on the electrode surface [24].

Here, we focused only on the analytical use of a scanning laser beam that vaporizes the surface material under flowing helium at atmospheric pressure. Such conditions should be fit for purpose without compromising bismuth stability. Although this technique possesses a low resolution, it has been already used for many applications for solid material characterization, giving information on the surface topology and on the qualitative and quantitative elemental composition [25,26]. We demonstrate here that the application of this technique is also suitable in the case of the analysis of bismuth films on screen-printed electrodes for electroanalysis. Laser ablation ICP-MS can permit a fast and stable measurement of the distribution of this metal on the electrode, with the advantage of a reduced alteration of the sample surface, avoiding possible interferences.

## 2. Materials and Methods

### 2.1. Apparatus and Reagents

Screen-printed electrodes (SPEs) have been a kind gift by Prof. Giuseppe Palleschi and his group at the University of Roma “Tor Vergata”. The printing procedures of the electrodes on a flexible polyester film have been described in the literature [27,28,29].

**Figure 1:**
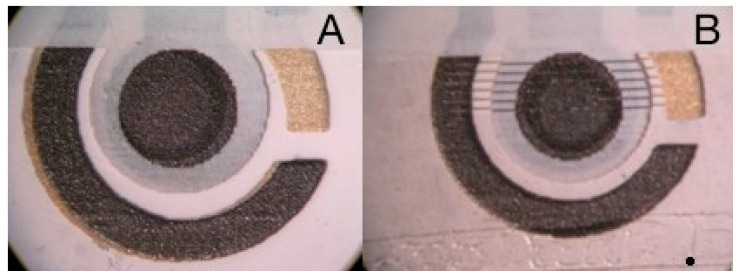
Optical micrographs of a freshly prepared, untreated Bi/PO_4_/PSS electrode before (**A**) and after (**B**) the laser ablation ICP-MS experiment.

An optical microscopy view of the electrode is shown in Figure 1A, where its three-electrode structure is evidenced. The carbon working electrode is in the center, with a diameter of 0.3 cm, along with the silver pseudo-reference electrode on the top right, and the surrounding carbon counter-electrode on the left.

Hydrochloric and nitric acid were TraceSelect from Fluka, and they were purified by sub-boiling distillation in a quartz-Teflon apparatus [30]. Chemical reagents (disodium hydrogen phosphate, potassium nitrate, sodium sulphate) were purchased from Aldrich. Poly (sodium-4-styrene sulfonate) (PSS, Aldrich, St. Louis, MO, USA) was used as such.

Standard stock solutions of Cd and Pb were purchased from Aldrich, and bismuth stock solution was prepared from bismuth nitrate pentahydrate (CarloErba). These 1000-mg/L solutions were diluted as required for the preparation of standards.

For standards and solutions, as well as glassware cleaning, high-purity MilliQ water from MilliPore was used.

### 2.2. Preparation of Bismuth Films Screen-Printed Electrodes from Insoluble Bismuth Phosphate

Pristine screen-printed electrodes have been used as received after cleaning in HCl solution. Polystyrene sulfonate (PSS)-modified Bi/PO_4_ electrodes were prepared via deposition of the polymer layer and of the insoluble bismuth phosphate, following the 2-step recipe in Reference [13]:

Step 1: 2 μL of a 25 mM solution of PSS is drop-casted onto the working electrode with a microsyringe and left for drying.

Step 2: equal volumes of Na_2_HPO_4_ (4 mM) and Bi(NO_3_)_3_ (2 mM) were mixed, and 5 μL of this solution is then drop-casted onto the working electrode and left for drying. These electrodes can be stored in air at room temperature.

Prior of their use for the electrochemical analysis, the active bismuth film is formed by cycling eight times in 0.01 M HCl (pH = 2) under the electroanalytical conditions for the differential pulse stripping analysis described below in Section 2.3. The activated electrodes must be stored in a diluted acid solution without contact to air.

For the nitrate or sulphate interference study, sodium sulphate or potassium nitrate were added equimolar to 0.01 M HCl during repetitive scan experiments.

### 2.3. Measurement Procedures

PalmSens (Palm Instrument BV, Houten, The Netherlands) and Amel4330 (Amel srl, Milano, Italy) instruments were used. All instrument control as well as the full data analysis were handled by the Windows-based VApeak software.

A special cell was designed in collaboration with Amel, in order to optimize mass transport to the electrodes. To avoid the drawbacks of the internal pseudo-reference electrode, an external 3 M KCl Ag/AgCl reference electrode was used in order to reference all potentials.

The analysis of cadmium and lead was performed in 0.01 M HCl, or 0.01 M HNO_3_, using differential pulse stripping voltammetry (DPV). High-purity nitrogen for degassing and blanketing was used throughout the entire stripping experiment. Both the activation of the electroactive bismuth film and the stripping analysis were done under the sane conditions. Potential was initially set at −1.05 V for 60 s at a stirring rate of 300 rpm; then, stirring was stopped, and the DPV analysis started, scanning the potential from −0.95 to −0.25 V, with a step potential of 5 mV, pulse time of 100 ms, and pulse amplitude of 50 mV.

Peak areas measured as (μA × mV) were used for quantitation. The best-fit linear calibration line was calculated as y = mx + q. Analytical sensitivity was defined as the slope (m) of the best-fit calibration line. Limits of detection (LOD) were calculated as 3σ/m and reported as μg/L. σ was determined from the standard deviation of 10 replicate analyses on a standard Pb and Cd sample with concentrations near the limit of detection.

### 2.4. LA-ICP-MS Analysis

An inductively coupled plasma-mass spectrometry (ICP-MS), model Thermo Scientific ICAP Q, coupled with a fourth harmonic Nd: YAG 266 nm laser system, model New Wave UP Series 266, was used for the laser ablation-ICP-MS (LA-ICP-MS) measurements. The sample chamber for laser scanning (a viscous film sample chamber was employed in this work, see reference [31] for details concerning the design and analytical performances) was flushed with high-purity helium, that was then interfaced with the ICP torch. A careful setting of the instrumental parameters has been done in order to optimize the mass signal, in terms both of speed and signal-to-noise ratio. The optimized parameters of the laser ablation ICP-MS instrument are reported in Table 1.

Optical microscopy views of the screen-printed electrode prior and after a full laser ablation scan are reported in Figure 1A,B, respectively. The six laser scans are clearly visible as thin dark lines. During the laser scan, the ablated material is transported by the helium flow into the ICP torch, and mass signals at ^13^C, ^31^P, and ^209^Bi are continuously recorded using a dwell time of 40 ms. The ^13^C signal is used as background to reference phosphorous and bismuth *m*/*z* signals in order to improve the signal stability and signal-to-noise ratios.

## 3. Results

### 3.1. Electrode Preparation

The concentration of bismuth in the pre-deposited BiPO_4_ phase as a precursor of the active Bi^0^ metal phase in screen-printed electrodes is known to have a profound effect on analytical performances. In both References [13,17], the best performances are shown by the lowest Bi concentration, with an ideal value around 1 mM in terms of peak shape and increased linearity at low analyte concentrations. For laser ablation studies, instead, the need to increase bismuth loadings was immediately evident, with respect to the best working low-loading electrode, in order to increase signal-to-noise ratios. Therefore, in this paper, bismuth film electrodes have been prepared and studied using an intermediate concentration of bismuth, namely 2 mM, trying to find a good compromise between analytical performance and S/N ratios in ICP-MS.

**Figure 2:**
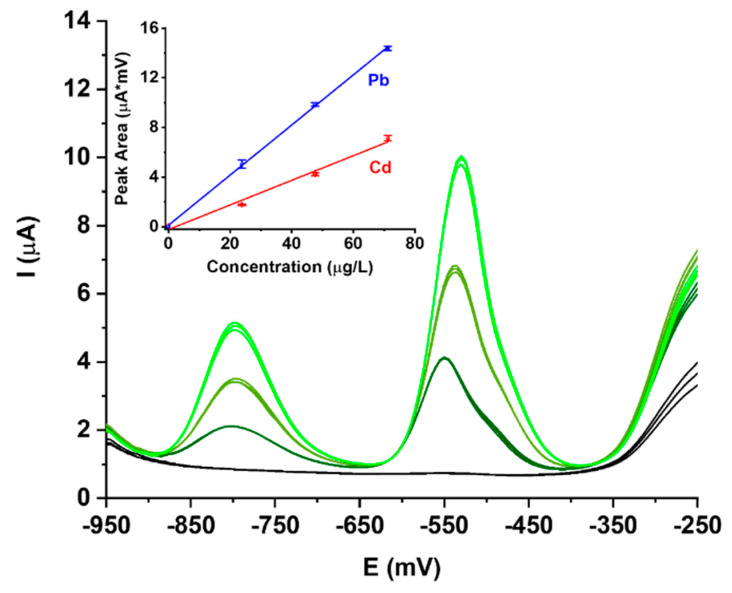
Differential pulse, anodic stripping voltammetry analyses of Cd and Pb solutions, and corresponding calibration curves (inset) at 0, 25, 50 and 75 μg/L on a working Bi/PO_4_/PSS electrode.

These bismuth film electrodes (BiFE) have been tested in the trace analysis of Cd and Pb under differential pulse stripping and the results are reported in Figure 2.

Baselines are very reproducible and free of noise; peaks are found at the expected potential, i.e., around −800 mV for Cd and around −530 mV for Pb. They show only a minor broadening observed as a shoulder, which is slightly more evident for Pb. For this reason, calibration curves are done on the integrated signals.

Calibration curves are very straight, and limits of linearity were not reached at the maximum concentration of 75 μg/L used in the calibration. Low-concentration nonlinearity is negligible and it can be seen by the small deviation of the X-intercept from zero (see inset in Figure 2). The non-linearity of bismuth film electrodes is well-known but does not significantly affect the limits of detection (LODs) for Cd and Pb which are in the μg/L range around 1.2 μg/L for Cd and 0.9 μg/L for Pb. These results compare well with those reported for BiPO_4_-derived BiFEs.

The stability of the bismuth phase has been checked by running a replicate analytical scan of Pb and Cd at 15 μg/L and checking baselines after each repetition. Baselines are always stable and free of noise, and the integrated analytical signals are not significantly decreased after 20 replicates. This is in full agreement with those previously reported, and, therefore, this is not reported here.

**Figure 3:**
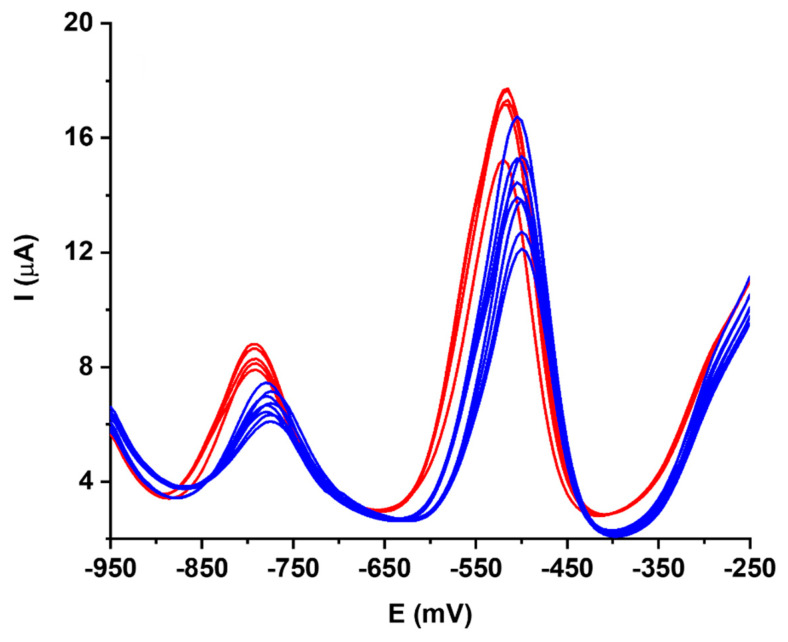
Effect of nitrate addition (blue trace) on Pb and Cd analysis in HCl at pH = 2 (red trace) on a working Bi/PO_4_/PSS electrode.

The effect of anions different from chloride in the matrix has been studied, in order to understand whether residual phosphate ions after activation may affect the analytical performance. Adding nitrate ions during a repetitive scan indicated a small shift in the peak maxima of about +20 mV and a small, but significant, loss of signal of about 10% (Figure 3).

**Figure 4:**
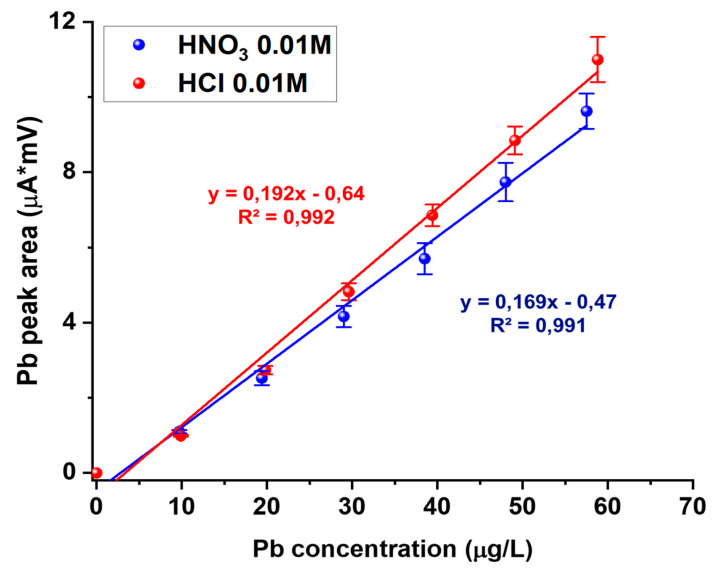
Comparison between calibration lines for Pb analysis in 0.01 M HCl (red line) and 0.01 M HNO_3_ (blue line) on a working Bi/PO_4_/PSS electrode.

This effect is then linked to a decrease in the analytical sensitivity, as evidenced in Figure 4 by comparing the calibration lines for Pb analysis in HCl or HNO_3_ matrix solutions at pH = 2. A similar effect was found with sulphate ion (data not shown here).

Therefore, a detailed characterization was thought to be interesting, both for investigating the structure of the active bismuth layer as well as for fully understanding the chemistry occurring during the reductive activation of the bismuth salt layer upon activation and electrochemical analysis, to understand whether phosphate ions are fully removed prior the analysis, or are still playing a role in the electrochemical analysis.

### 3.2. Laser Ablation-ICP-MS Characterization

The characterization of bismuth film deposited onto the screen-printed working electrode was carried out by scanning the high-power UV laser beam through the *X*-axis of the dry electrode, while the ablated material is analyzed in real time by ICP-MS.

**Figure 5:**
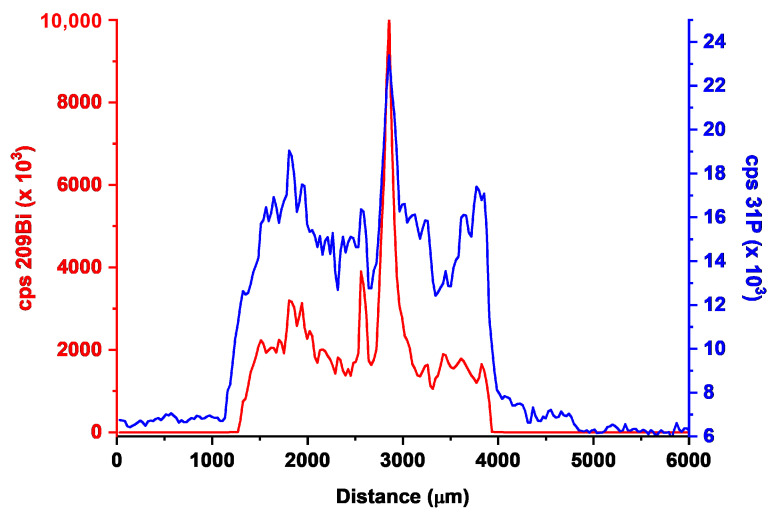
Comparison of the 209 Bi (red line) and 31P (blue line) laser ablation profiles located at 260 µm on a freshly prepared, untreated Bi/PO_4_/PSS electrode.

The analysis is first performed on an untreated screen-printed electrode right after the preparation of the BiPO_4_ layer via drop-casting deposition onto the carbon working electrode. The first result on a single scan line is reported in Figure 5, where the traces corresponding at the two isotopes of ^209^Bi and ^31^P are compared. The signal is, however, a little bit noisy, and a 10-point moving average smoothing is routinely applied throughout the paper.

It is immediately evident that the background signal for ^209^Bi is very low, since no bismuth is expected to be present on the pristine (untreated) screen-printed electrode. The ^209^Bi signal is almost perfectly confined at the center of the scan, which corresponds exactly to the borders of the carbon working electrode, with minimal spreading outside (please note in Figure 5 that the ^209^Bi signal is appreciable for a length of 0.3 cm, i.e., the diameter of the working electrode). This may demonstrate that the careful choice of the drop-casting procedure may lead to the preferential formation of the BiPO_4_ phase on the carbon surface. Obviously, this may be helped by the hydrophobic character of the plastic surface of the screen-printed electrode (SPE), preventing unwanted spreading during evaporation of the two subsequent layers of PSS and Na_2_HPO_4_ + Bi(NO_3_)_3_ solution_._

Looking in more detail at the concentration profile of ^209^Bi, the presence of peaks and valleys reveals the inhomogeneous nature of the Bi distribution over the working electrode. Since the intensity signal is essentially correlated with the thickness of the bismuth layer onto the surface, the irregular shape of the bismuth signal may suggest an uneven Bi distribution with the presence of much thicker aggregates giving the most intense signals.

It is interesting to compare the traces of ^209^Bi and ^31^P on the same scanning line. The phosphorous signal is much less intense than the bismuth one (please note that different scales were used for ^209^Bi and ^31^P signals in Figure 5): this is a result of the much lower sensitivity of *m*/*z* = 31 of phosphorous with respect to the *m*/*z* = 209 signal of bismuth, which is characterized by a very high sensitivity. Secondly, some phosphorous has to be pristinely present in the polymeric base of the electrode, leading to high background values. Both facts have the consequence that the ^31^P signal shows low S/N ratios, and its plots are intrinsically less diagnostic than ^209^Bi. Thirdly, it is noteworthy to evidence that the concentration profile of ^31^P closely resembles that of ^209^Bi, although it is less defined as said before. This is a clear indication that BiPO_4_ is the prevailing chemical form present on the electrode surface, with only a residual presence of Bi-rich or P-rich phases which could be formed upon evaporating the aqueous impregnation solution of Na_2_HPO_4_ and Bi(NO_3_)_3_.

**Figure 6:**
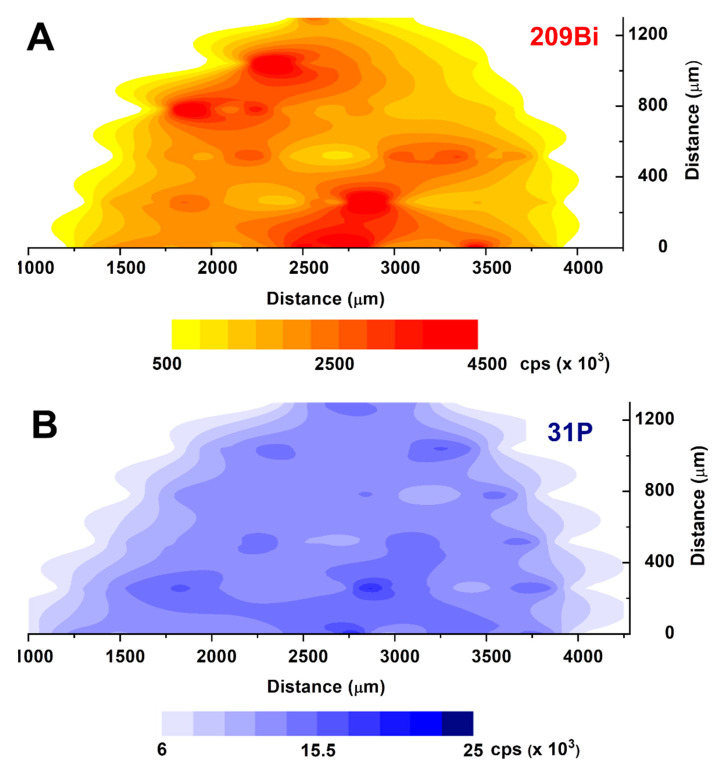
LA-ICP-MS contouring plots for 209Bi ((**A**), **top**) and 31P ((**B**), **bottom**) on a freshly prepared, untreated Bi/PO_4_/PSS electrode.

In Figure 6, the contouring plots of ^209^Bi (Figure 6A) and ^31^P (Figure 6B) are shown.

The spatial resolution is, unfortunately, quite poor due to the spot size of the laser beam which is necessary for having a reasonable signal for phosphorous. In any case, it can be immediately evidenced the presence of large aggregates of a microcrystal, where bismuth and phosphorous seem to be simultaneously present in the form of BiPO_4_ crystals.

**Figure 7:**
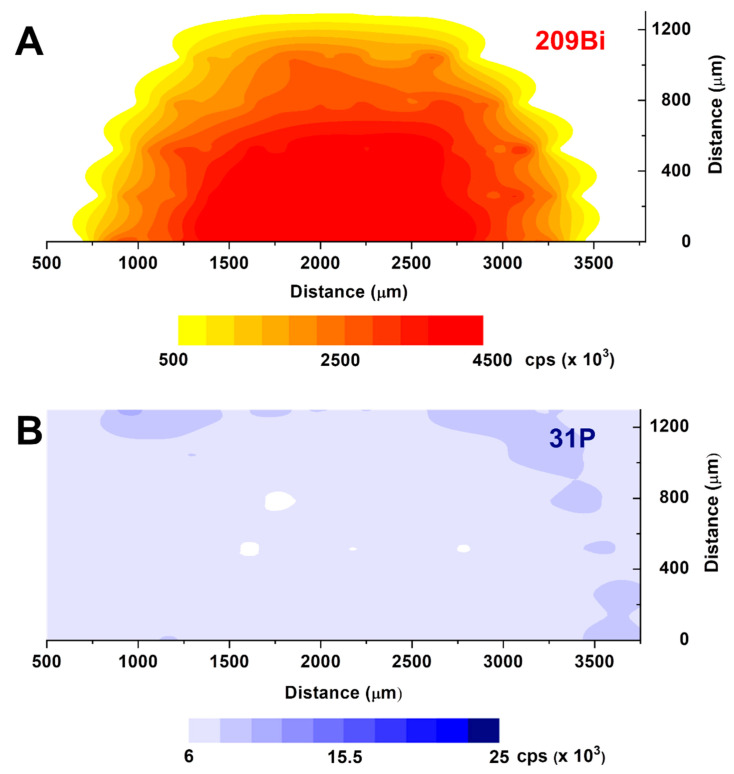
LA-ICP-MS contouring plots for 209Bi ((**A**), **top**) and 31P ((**B**), **bottom**) on a working Bi/PO_4_/PSS electrode after electrochemical activation and real analysis.

The laser ablation ICP-MS results on the used electrodes after electrochemical activation are reported in Figure 7 and show a totally different chemical and structural situation.

In fact, the plot at *m*/*z* = 209 (Figure 7A) shows a pretty regular, spherical distribution of bismuth, with the maximum concentration on the center of the graphite surface of the working electrode. Instead, the phosphorous signal at *m*/*z* = 31 of Figure 7B is completely lost on the electrode surface, with some residual signals being only left at the borders, where the potential density during the electrochemical measurement is minimal. These results are very important, in that they confirm our previous hypothesis that no phosphorous should be left upon electrochemical activation of the BiPO_4_ surface. The possible effect of anions different from chloride, that was shown to slightly decrease the analytical sensitivity and peak intensity, is to be independent from the residual presence of phosphate, or phosphorous species, after electrode activation, as it was previously supposed. This is to be related to two possible causes: (i) to the different diffusivity of the anions within the PSS layer, or (ii) to the complexing properties of chloride ions, with respect to nitrate or sulphate. This second hypothesis is thought to be the prevailing factor, as it may be evidenced by the positive potential shift in the peak position of Pb and Cd. In fact, bismuth surface activation is due to a reductive elimination reaction when negative potential is applied, leading to the reduction of Bi(III) to Bi(0) and the parallel elimination of phosphate ions for the redox balance. The experimental conditions that have been chosen then allow the zerovalent bismuth phase to slowly grow, reaching its maximum thickness at the center of the working electrode. In fact, the kinetics of this reductive elimination process is mainly governed by the solid–liquid electrolyte interphase. This regular shape of the bismuth surface may also explain the good reproducibility and stability of the stripping analyses.

## 4. Conclusions

Our preliminary investigation has shown how laser ablation ICP-MS may play an important role in the characterization of electrochemical surfaces. In fact, the electrode surface is simply dried in a nitrogen atmosphere before placing it under the laser compartment under helium at atmospheric pressure. In this way, no contamination from air is possible, and no restructuring of the mobile bismuth (or mercury) surface can occur, as would be taking place for conventional techniques, such as scanning electron microscopy, using high-energy electron or photon beams under high-vacuum conditions.

This investigation clearly evidenced how the phosphate counteranion is completely lost upon reductive activation in the electrochemical cell, leading to a very clean, “ball-type” bismuth active surface. The formation of such a bismuth surface may account for any anion-type interference, as well as of the non-linearities at low analyte concentrations with increasing bismuth loadings.

Finally, these screen-printed electrodes, based on Brainina’s original ideas, are demonstrated to be viable alternatives to the in situ-prepared bismuth film electrodes. They are indefinitely stable in air, easily activated under the reductive conditions of the stripping electroanalytical experiment, and their final properties may be tuned by varying the bismuth concentration and deposition conditions.

## Figures and Tables

**Table 1:** List of instrumental parameters for the laser ablation (LA)-ICP-MS experiments.

Laser Ablation Parameters	
Scan Speed	20 μm/s
% output	20%
Step rate	20 Hz
Laser spot size	80 μm
Distance between each laser scan	260 μm
**Mass Spectrometer Parameters**	
Dwell Time	40 ms
PC Detector	3300
